# Supplementary material for: Modeling glioblastoma heterogeneity as a dynamic network of cell states
Source: Mol Syst Biol. 2021 Sep 16;17(9):e10105. doi: 10.15252/msb.202010105 (PMC8444284; doi:10.15252/msb.202010105)
Supplement: Supplementary file 6 — Source Data for Figure 5 [file MSB-17-e10105-s004.zip › Figure5A_sourcedata/GSEA_3017/nosigdb_stateA.GseaPreranked.1622795026485/LARSSON_STATE_4.html]

Details for gene set LARSSON\_STATE\_4[GSEA]

|  || Dataset | state53017 |
| Phenotype | NoPhenotypeAvailable |
| Upregulated in class | na\_pos |
| GeneSet | LARSSON\_STATE\_4 |
| Enrichment Score (ES) | 0.4708078 |
| Normalized Enrichment Score (NES) | 3.0106542 |
| Nominal p-value | 0.0 |
| FDR q-value | 0.0 |
| FWER p-Value | 0.0 |
Table: GSEA Results Summary

  

Fig 1: Enrichment plot: LARSSON\_STATE\_4      
 Profile of the Running ES Score & Positions of GeneSet Members on the Rank Ordered List

  

| PROBE | GENE SYMBOL | GENE\_TITLE | RANK IN GENE LIST | RANK METRIC SCORE | RUNNING ES | CORE ENRICHMENT || 1 | CDK1 |  |  | 3 | 1.006 | 0.0388 | Yes |
| 2 | MKI67 |  |  | 4 | 0.926 | 0.0775 | Yes |
| 3 | UBE2C |  |  | 7 | 0.793 | 0.1084 | Yes |
| 4 | MCM10 |  |  | 9 | 0.752 | 0.1388 | Yes |
| 5 | KPNA2 |  |  | 20 | 0.667 | 0.1559 | Yes |
| 6 | HIST1H4C |  |  | 21 | 0.659 | 0.1834 | Yes |
| 7 | PCLAF |  |  | 28 | 0.637 | 0.2036 | Yes |
| 8 | CCNB1 |  |  | 37 | 0.599 | 0.2200 | Yes |
| 9 | CKS2 |  |  | 40 | 0.587 | 0.2423 | Yes |
| 10 | NCAPG |  |  | 44 | 0.556 | 0.2624 | Yes |
| 11 | CKS1B |  |  | 50 | 0.530 | 0.2791 | Yes |
| 12 | DBF4 |  |  | 55 | 0.514 | 0.2963 | Yes |
| 13 | TPX2 |  |  | 66 | 0.488 | 0.3059 | Yes |
| 14 | MYBL2 |  |  | 69 | 0.486 | 0.3241 | Yes |
| 15 | SGO2 |  |  | 73 | 0.478 | 0.3408 | Yes |
| 16 | HIST1H1B |  |  | 77 | 0.475 | 0.3574 | Yes |
| 17 | UBE2T |  |  | 83 | 0.470 | 0.3717 | Yes |
| 18 | DUT |  |  | 87 | 0.466 | 0.3879 | Yes |
| 19 | CENPF |  |  | 94 | 0.461 | 0.4008 | Yes |
| 20 | TOP2A |  |  | 95 | 0.457 | 0.4198 | Yes |
| 21 | HIST1H1D |  |  | 98 | 0.456 | 0.4367 | Yes |
| 22 | ZWINT |  |  | 106 | 0.447 | 0.4479 | Yes |
| 23 | SGO1 |  |  | 126 | 0.425 | 0.4452 | Yes |
| 24 | PIMREG |  |  | 143 | 0.413 | 0.4453 | Yes |
| 25 | GTSE1 |  |  | 146 | 0.411 | 0.4603 | Yes |
| 26 | MAD2L1 |  |  | 153 | 0.406 | 0.4708 | Yes |
| 27 | MCM7 |  |  | 171 | 0.394 | 0.4690 | No |
| 28 | PTMA |  |  | 206 | 0.371 | 0.4480 | No |
| 29 | SMC4 |  |  | 217 | 0.367 | 0.4526 | No |
| 30 | H2AFZ |  |  | 245 | 0.351 | 0.4383 | No |
| 31 | TUBB |  |  | 264 | 0.340 | 0.4331 | No |
| 32 | CLSPN |  |  | 274 | 0.336 | 0.4375 | No |
| 33 | ESCO2 |  |  | 282 | 0.333 | 0.4439 | No |
| 34 | ORC6 |  |  | 283 | 0.333 | 0.4578 | No |
| 35 | MND1 |  |  | 304 | 0.324 | 0.4498 | No |
| 36 | CENPN |  |  | 310 | 0.323 | 0.4579 | No |
| 37 | FANCI |  |  | 319 | 0.319 | 0.4627 | No |
| 38 | ANP32B |  |  | 357 | 0.307 | 0.4357 | No |
| 39 | BRCA1 |  |  | 373 | 0.302 | 0.4323 | No |
| 40 | RPA3 |  |  | 381 | 0.300 | 0.4373 | No |
| 41 | RRM2 |  |  | 383 | 0.300 | 0.4487 | No |
| 42 | PHF19 |  |  | 393 | 0.297 | 0.4514 | No |
| 43 | HMGB1 |  |  | 403 | 0.292 | 0.4540 | No |
| 44 | HMGB2 |  |  | 424 | 0.288 | 0.4445 | No |
| 45 | NUSAP1 |  |  | 430 | 0.286 | 0.4511 | No |
| 46 | LMNB1 |  |  | 442 | 0.282 | 0.4510 | No |
| 47 | CDC45 |  |  | 446 | 0.282 | 0.4596 | No |
| 48 | ATAD2 |  |  | 470 | 0.275 | 0.4464 | No |
| 49 | EZH2 |  |  | 471 | 0.275 | 0.4579 | No |
| 50 | KIF4A |  |  | 504 | 0.269 | 0.4347 | No |
| 51 | UBE2S |  |  | 507 | 0.269 | 0.4438 | No |
| 52 | ALYREF |  |  | 535 | 0.264 | 0.4258 | No |
| 53 | RANBP1 |  |  | 536 | 0.263 | 0.4368 | No |
| 54 | KIF15 |  |  | 538 | 0.263 | 0.4467 | No |
| 55 | CDKN3 |  |  | 539 | 0.263 | 0.4577 | No |
| 56 | FBXO5 |  |  | 557 | 0.258 | 0.4502 | No |
| 57 | TMPO |  |  | 584 | 0.253 | 0.4329 | No |
Table: GSEA details [plain text format]

  

Fig 2: LARSSON\_STATE\_4: Random ES distribution      
 Gene set null distribution of ES for **LARSSON\_STATE\_4**
